# Postmortem plasma pentraxin 3 is a useful marker of fatal acute coronary syndrome

**DOI:** 10.1038/s41598-019-44472-0

**Published:** 2019-05-30

**Authors:** Misa Tojo, Kaori Shintani-Ishida, Hajime Tsuboi, Mami Nakamura, Nozomi Idota, Hiroshi Ikegaya

**Affiliations:** 1 Department of Forensic Medicine, Graduate School of Medical Science, Kyoto Prefectural University of Medicine, Kyoto, Japan; 2 Present Address: Department of Legal Medicine, Shiga University of Medical Science, Shiga, Japan

**Keywords:** Biomarkers, Cardiology

## Abstract

Pentraxin 3 (PTX3) is an acute-phase protein that belongs to the pentraxin superfamily. Recently, many clinical studies have demonstrated that plasma PTX3 concentrations rapidly increase in patients with the acute coronary syndrome (ACS). The aim of this study was to evaluate the forensic utility of postmortem plasma PTX3 as a marker of fatal ACS. We compared the plasma PTX3 concentration in cadavers with suspected fatal ACS to that exhibited in control cases (e.g., asphyxia and immediate death due to a fatal injury). The ACS groups included a coronary stenosis group, which exhibited apparent coronary stenosis, but an absence of coronary thrombi, a coronary thrombi group with thrombi found in the coronary artery, and a group of myocardial rupture following an acute myocardial infarction. The plasma PTX3 concentration was significantly higher in the coronary thrombi group than the control group and other ACS groups. The postmortem plasma PTX3 concentration was higher than the clinical reference values, which appeared to be caused by a postmortem release from circulating neutrophils. In conclusion, although the clinical reference value cannot be applied to postmortem samples, the postmortem plasma PTX3 concentration may be a useful marker of death occurring immediately after the onset of fatal ACS.

## Introduction

Most sudden cardiac deaths are caused by acute coronary syndromes (ACS) which include unstable angina pectoris (UAP) and acute myocardial infarction (AMI). Histopathological diagnosis of fatal ACS is challenging to obtain, as the pathological hallmarks of AMI, such as coagulative necrosis, “wavy fibers,” splitting, and contraction band necrosis^1,2^ are non-specific. Also, biomarkers of myocardium damage, including creatine kinase-MB (CK-MB) isoforms and cardiac troponins, remain unaltered for only a few hours after the onset of AMI. This is because the plasma CK-MB and troponin concentrations only begin to increase between 3–8 h and 4–6 h, respectively, after the onset of an AMI^3^. A recent report demonstrated that a high-sensitivity cardiac troponin I assay could evaluate the risks of subsequent AMI and cardiac death in patients with suspected ACS^4^. However, during a postmortem examination, these enzymes present in cardiomyocytes should be used with caution due to the postmortem release into blood^5^.

In a forensic autopsy, the degree of coronary stenosis is commonly determined to be a possible culprit site of ACS. However, it is generally accepted that fatal ACS is not associated with the progression of the luminal stenosis to a critical degree of narrowing, but rather the rupture of an atherosclerotic plaque^6,7^. An erosion or rupture of the plaque triggers fatal ACS only when coinciding with thrombus formation at the site of plaque erosion or rupture. However, during a forensic autopsy, it is tough to detect the culprit thrombi remaining in the postmortem coronary artery. During plaque formation, local inflammation occurs, which involves neutrophils, macrophages, and other immune-competent cells^8^. Several studies have demonstrated that markers of systemic inflammation, including C-reactive protein (CRP)^9,10^, serum fibrinogen^10^, leukocyte count^11^, and cytokines^12^ are increased in the plasma concentration of patients with atherosclerosis, especially in those with unstable coronary artery diseases. However, the systemic inflammation in which these markers participate is not sensitive enough to quickly respond to a sudden plaque rupture and the subsequent dramatic formation of an occlusive thrombus. For example, Peri *et al*. reported that the CRP plasma concentration in patients admitted to the coronary care unit (CCU) with AMI symptoms was not elevated at the time of entry to the CCU, finally became raised at 12 h, and peaked at 24 h^13^.

Recently, pentraxin 3 (PTX3), a component of the same pentraxin superfamily as CRP, has attracted attention as a rapid and sensitive biomarker of ACS, including UAP^14–16^ and AMI^13^. CRP and serum amyloid P component (SAP), another pentraxin family member, are created primarily in the liver in response to the inflammatory mediator interleukin (IL)-6. In contrast, PTX3 is induced locally at the site of inflammation, but not within the liver. Moreover, PTX3 is secreted following Toll-like receptor activation and proinflammatory cytokine secretion, including tumor necrosis factor-α and IL-1, but not IL-6 (reviewed by^17^). Therefore, the induction of PTX3 is more rapid than CRP or SAP. In the CCU administration cases described above, the plasma PTX3 concentration in AMI patients was already elevated at the time of entry to the CCU and reached a peak at 7.5 h^13^. In addition, because the induction of PTX3 is highly sensitive to local inflammation, the plasma PTX3 concentration in patients with vascular atherosclerosis is likely reflective of the inflammatory status of the vasculature (i.e., the presence of plaque formation). In support of this concept, it was reported that the plasma PTX3 concentration increased in patients with high-risk plaque components^15^ and with UAP, but not in those with effort angina^14^.

In the present study, we evaluated the forensic utility of the postmortem plasma PTX3 concentration as a biomarker for fatal ACS in cadavers. We compared the plasma PTX3 concentration in forensic autopsy cases of ACS with that in control cases. As inflammation-positive controls, serious infection cases were also investigated.

## Results

### Plasma PTX3 and CRP concentrations by the cause of death

**Figure 1:**
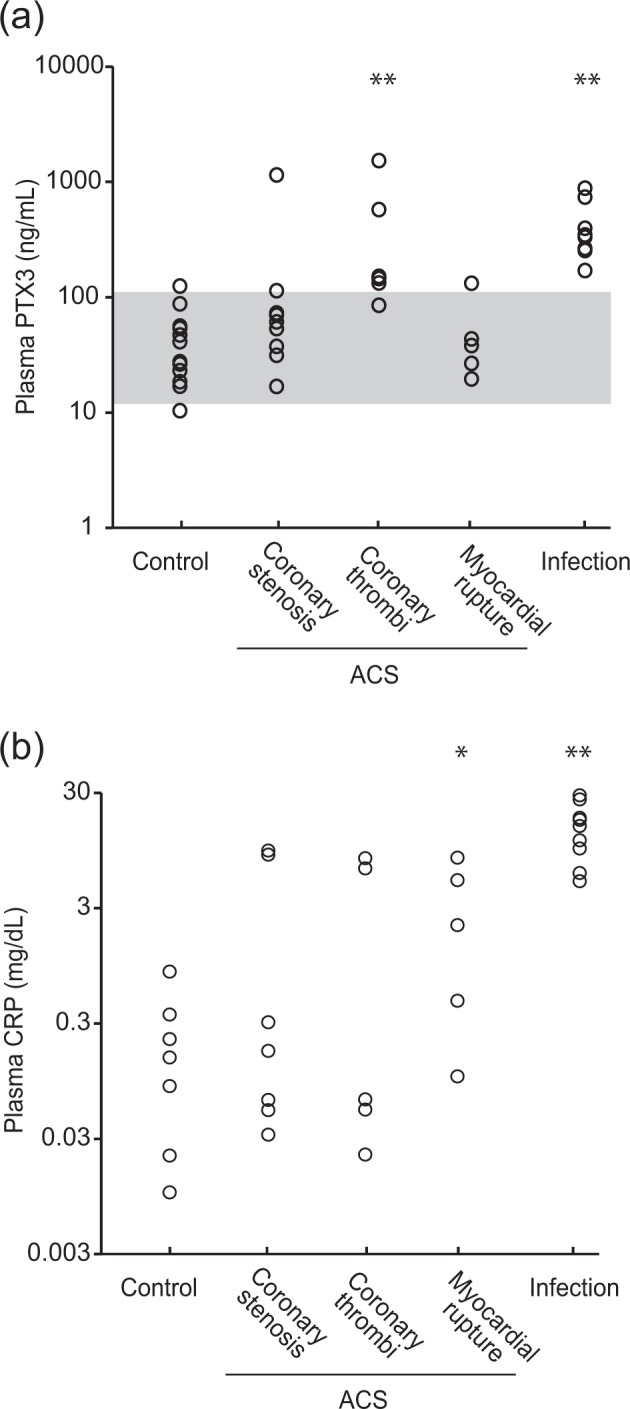
Plasma PTX3 and CRP concentrations by cause of death. Panel a shows plasma PTX3 concentration. The gray zone indicates the 95% prediction interval of the control group (11.96–110.0 ng/mL). Panel b shows plasma CRP concentration. ACS, acute coronary syndrome. *<0.05; **<0.01.

The plasma PTX3 concentration in the control group was less than 120 ng/mL (43.0 ± 32.0 ng/mL) (Fig. 1a, Table 1). Seven out of nine cases of the coronary stenosis group and 4 out of 5 cases of the myocardial rupture group in the ACS groups exhibited a plasma PTX3 concentration less than 110.0 ng/mL, which is the 97.5th percentile in the control group. Conversely, in the coronary thrombi cases of the ACS group, 5 out of 6 cases exhibited a plasma PTX3 concentration higher than 110.0 ng/mL. In the serious infection group, all cases exhibited a high concentration of PTX3 (462.1 ± 266.6 ng/mL). A multiple-comparison analysis (Steel test following to Kruskal–Wallis test) among these groups determined that the coronary thrombi group and serious infection group were significantly different from the control group (*P* = 0.0077 and 0.0003, respectively).

**Table 1:** Case profiles and assay results.

Case No.	Age (years)	Sex	Postmortem Interval (h)	Plasma PTX3 (ng/mL)	W-LCC (×10^2^/μL)	Plasma CRP (mg/dL)	Drug Screening
**Control (** ***N*** ** = 12)**							
1	62	Female	41	26.71	34	0.02	—
2	53	Male	34	10.29	20	0.2	—
3	63	Female	26	45.37	69	0.02	Chlorpheniramine
							Lidocaine
4	88	Male	39	54.74	65	0.01	Metoclopramide
							Paroxetine
							Donepezil
							Domperidone Cimetidine
5	30	Male	26	84.17	86	0.01	Lidocaine
6	67	Male	23	40.27	25	0.74	Chlorpheniramine
							Paracetamol
							Dextromethorphan
							Methylephedrine
7	74	Female	24	119.9	46	0.01	Chlorpheniramine
							Paracetamol
							Dextromethorphan
							Methylephedrine
8	54	Male	35	17.96	37	0.02	—
9	71	Male	36	16.35	—	0.02	Pheniramine
10	40	Male	35	53.02	—	0.32	Lidocaine
11	83	Male	22	25.67	20	0.14	Chlorpheniramine
							Lidocaine
12	54	Male	37	22.11	14	0.08	—
	61.6 ± 16.7	9/3^a^	31.5 ± 6.8	43.04 ± 32.00	41.6 ± 24.4	0.133 ± 0.215	
**ACS: coronary stenosis (** ***N*** ** = 9)**							
13	69	Female	37	1102	—	8.14	Mirtazapine
							Amiodarone
14	83	Male	38	59.71	10	0.06	—
15	71	Female	24	31.32	31	7.46	Lidocaine
16	74	Male	68	52.66	—	0.03	—
17	79	Male	23	16.47	55	0.05	—
18	53	Male	35	69.83	76	n.d.	—
19	42	Male	35	37.44	46	0.28	—
20	41	Male	5	69.18	—	0.16	—
21	66	Male	66	112.3	17	0.05	Amiodarone
	64.2 ± 15.4	7/2^a^	36.8 ± 20.0	172.3 ± 349.6	39.2 ± 24.8	2.03 ± 3.57	
**ACS: coronary thrombi (** ***N*** ** = 6)**							
22	74	Female	44	1380	57	6.95	Warfarin
23	47	Male	68	134.2	—	0.05	—
24	47	Male	48	140.23	85	0.02	—
25	70	Male	69	527.2	42	n.d.	—
26	39	Female	30	77.98	—	0.06	—
27	75	Female	44	119.4	—	5.5	—
	58.7 ± 16.1	3/3^a^	50.5 ± 15.2	396.5 ± 509.6	61.3 ± 21.8	2.52 ± 3.42	
**ACS: myocardial rupture (** ***N*** ** = 5)**							
28	71	Male	33	42.64	71	0.39	—
29	61	Female	42	36.08	82	1.77	—
30	68	Male	49	26.13	—	4.25	n.d.
31	62	Male	23	18.92	19	6.67	—
32	62	Male	21	125.1	58	0.09	Zolpidem
							Lidocaine
	64.8 ± 4.4	4/1^a^	33.6 ± 12.0	49.78 ± 43.10	57.5 ± 27.5	2.63 ± 2.79	
**Infection (** ***N*** ** = 10)**							
33	79	Male	29	169.6	111	22.9	—
34	80	Male	15	353.1	20	13.6	Donepezil
							Lidocaine
35	48	Female	44	354.9	—	15.2	Paroxetine
							Zopiclone
							Tramadol
							Cetirizine
							Diclofenac
							Methaqualone
36	74	Female	72	877.6	11	25.2	—
37	86	Female	16	264.6	98	4.64	—
38	53	Male	10	395	117	8.66	—
39	75	Male	30	254.7	—	5.37	Warfarin
40	65	Male	39	328.8	—	16.1	Verapamil
							Lidocaine
41	41	Male	39	880.2	23	41.7	—
42	84	Male	46	742.9	220	10.4	—
	66.8 ± 15.9	7/3^a^	33.7 ± 18.3	462.1 ± 266.6	85.7 ± 74.9	16.4 ± 11.2	

Statistical data denote the mean ± SD. ^a^Male/female; W-LCC, white blood cell-large cell counts; — undeterminable (W-LCC) or undetected (drug screening); n.d., not done.

Contrary to the plasma PTX3 concentration, the plasma CRP concentration in the ACS groups was significantly higher not in the coronary thrombi group but in the myocardial rupture group (Fig. 1b, Table 1). In the coronary thrombi group, half of the cases had a plasma CRP concentration less than 0.3 ng/mL, which is the upper clinical range limit, whereas in the myocardial rupture group, 4 out of 5 cases were over 0.3 mg/dL. In the serious infection group, all cases exhibited a high concentration of CRP, as well as PTX3 concentration.

### Postmortem effects on the plasma PTX3 concentration

**Figure 2:**
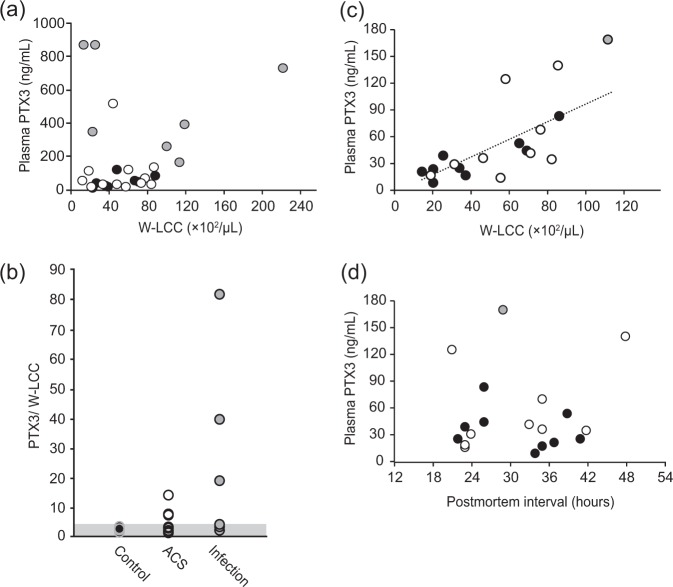
The relationship between plasma PTX3 concentration and circulating neutrophil counts or postmortem intervals. Panel a shows the relationship between plasma PTX3 concentration and white blood cell-large cell counts (W-LCC) values. Panel b shows the ratios of the PTX3 concentration to W-LCC by cause of death. The gray zone indicates the 95% prediction interval of the control groups (0.492–2.38). Panels c and d denote the relationship between plasma PTX3 concentration and W-LCC or postmortem intervals in cases with low PTX3/W-LCC ratios (<2.38). Black circles, control group; white circles, acute coronary syndrome (ACS) groups; gray circles, infection group.

**Figure 3:**
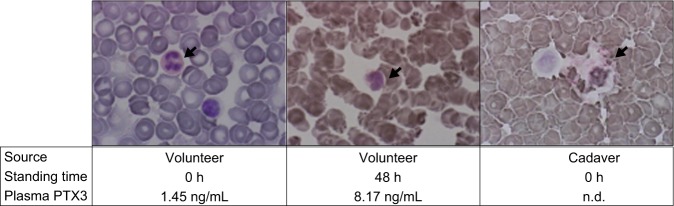
Representative images of PTX3-immunostained neutrophils in the smear samples of fresh blood collected from a healthy volunteer, the *in vitro* stored blood for 48 h, or blood from a cadaver with postmortem interval at about 16 h. PTX3-positive neutrophils (the left image) were stained pink with New Fuchsin. The arrows show neutrophils. n.d., not done

**Figure 4:**
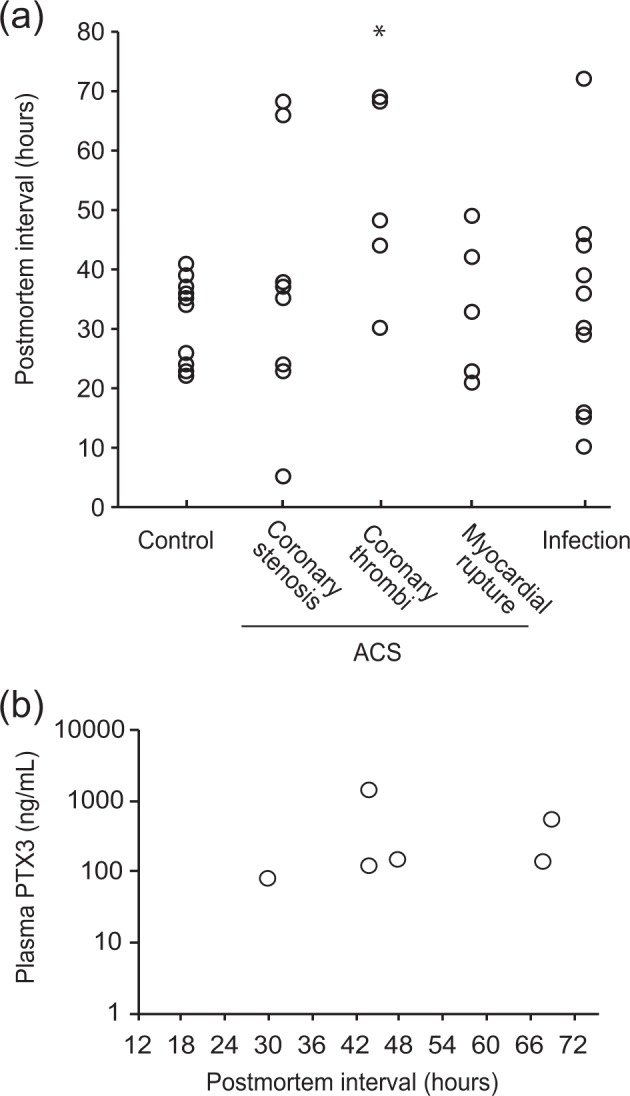
The relationship between plasma PTX3 concentration and postmortem intervals in the coronary thrombi group of the acute coronary syndrome (ACS) groups. Panel a shows the postmortem interval by cause of death. *<0.05. Panel b shows the relationship between plasma PTX3 concentration and the postmortem interval in coronary thrombi group.

The postmortem plasma PTX3 concentration was substantially higher than the clinical reference values reported previously: 43.0 ± 32.0 ng/mL in our control groups versus 1.98 ng/mL for the healthy volunteers^14^. PTX3 is produced *de novo* in neutrophil precursors, stored in neutrophil granules, and released in response to inflammatory signals^18^. Compared with lymphocytes, eosinophils, and monocytes, neutrophils degenerate rapidly following death. Morphological changes (e.g., pyknosis, cytoplasmic and nuclear vacuolation) and disintegration in neutrophils begin as early as 6 h after death^19–21^. These findings led us to investigate the postmortem release of PTX3 from circulating neutrophils. There appeared to be a relationship between the neutrophil counts and plasma PTX3 concentration (Fig. 2a, Table 1). However, this relationship was only present in cases with a plasma PTX3 concentration less than 200 ng/mL. Such high relative PTX3 concentration to neutrophil count ratios were found in only a few cases of the ACS and infection groups (Fig. 2b, Table 1). Accordingly, cases with PTX3/neutrophil count ratios above the 97.5th percentile (2.38) in the control groups (1.13 ± 0.66) were excluded from the correlation analysis. As a result, there was a strong relationship between the plasma PTX3 concentration and neutrophil counts (*P* < 0.001, *r* = 0.744) (Fig. 2c). In contrast, the plasma PTX3 concentration did not correlate with the postmortem intervals (*P* = 0.7221) (Fig. 2d). Following immunostaining of the blood smear samples, the cytoplasm of the neutrophils in fresh blood collected from a healthy volunteer stained positive with an anti-PTX3 antibody (Fig. 3, left panel). The neutrophils in the blood that was incubated at room temperature for 48 h after collection from the same volunteer (Fig. 3, middle panel) and those in the blood derived from a cadaver with a postmortem interval of approximately 16 h (the right panel) exhibited the same morphological changes as previously reported, including pyknosis, cytoplasmic and nuclear vacuolation, nuclear fragmentation, and disintegration^19–21^. Moreover, although the PTX3 concentration in the plasma from the fresh blood was 1.45 ng/mL, the concentration from the blood stored *in vitro* for 48 h was as high as 8.17 ng/mL (Fig. 3). These results indicate that the plasma PTX3 concentration increases immediately after death via postmortem release from circulating neutrophils. In the ACS groups, the postmortem interval (Fig. 4a), as well as the plasma PTX3 concentration (Fig. 1a), were significantly higher in the coronary thrombi group compared with the control group. However, there was no relationship between the postmortem interval and the plasma PTX3 concentration in the coronary thrombi group (Fig. 4b, Table 1, *P* = 0.9844).

PTX3 also has an inverse relationship with the development of obesity, as the plasma PTX3 concentration has been reported to significantly decrease with an increasing BMI^22^. In the present study, we found no relationship between the postmortem plasma PTX3 concentration and BMI (*P* = 0.2303).

Plasma PTX3 concentration is reportedly normalized by cardiovascular drugs, including antihypertensive^23–27^, antilipemic^28^, and antithrombotic^29^ agents. Although the drug screening test detected the antithrombotic drug warfarin in two cases (case Nos. 22 and 39 in Table 1), there seemed to be no influence on the plasma PTX3 concentration.

## Discussion

The present study demonstrated that all cases of the coronary thrombi group exhibited a significantly higher plasma PTX3 concentration. Immunohistochemical analysis using an antibody against PTX3 revealed strong positive staining of PTX3 in advanced atherosclerotic plaques of the coronary artery^16,30,31^. In blood samples collected from sites distal to the plaque lesions, the concentration of PTX3 was significantly higher than that obtained from proximal sites^14^. This indicates that the increased circulating PTX3 is derived from atherosclerotic plaques. PTX3 is produced by macrophages^30,31^, endothelial cells^30^, and smooth muscle cells^30^ found within atherosclerotic lesions. Furthermore, PTX3 has been detected in neutrophils infiltrating into atherosclerotic plaques and in coronary arterial thrombi removed from a culprit lesion^31^. Conversely, all but one of the cases in the coronary stenosis group without coronary thrombi had a plasma PTX3 concentration not significantly different from the control group. Consistent with these findings, the plasma PTX3 concentration increases in patients with high-risk plaque components^15^ and with UAP, but not in those with effort angina^14^. It seemed that the coronary stenosis case with a high plasma PTX3 concentration failed to detect advanced atherosclerotic plaques.

Plasma PTX3 concentration significantly increased in the coronary thrombi group, whereas in the myocardial rupture group, it returned to levels similar to the control group. Contrary to plasma PTX3 concentration, plasma CRP concentration was less than 0.3 mg/dL, the upper clinical range limit, in 3 out of 5 coronary thrombi cases, whereas CRP concentration was higher than 0.3 mg/dL in 4 out of 5 myocardial rupture cases. The kinetics of plasma PTX3 differs from that of CRP in AMI patients. Specifically, PTX3 peaks at 7.5 h and returns to normal concentration by 48 h after the onset of an AMI, whereas the increase in CRP begins at 18 h and is sustained until hospital discharge (>96 h)^13^. In a murine model of an AMI involving coronary artery ligation/reperfusion, PTX3 expression was upregulated in the interstitium surrounding the necrotic myocardium at 8 h of reperfusion after ischemia^32^. In this model, the plasma PTX3 concentration was also found to be elevated at 8 h, peaking at 24 h, and followed by a rapid return to normal concentration levels by 48 h. Myocardial rupture following AMI generally occurs between a few hours and 48 h after an AMI^33^, and all cases of myocardial rupture in this study exhibited histopathological findings of inflammatory cell infiltration, indicating that death occurred later than 24 h post-AMI^34^. These results suggest that an increased plasma PTX3 concentration indicates that death occurred immediately after the onset of fatal ACS.

The plasma PTX3 concentration obtained from cadavers was substantially higher than that obtained from patients^13–15^. This is likely due to the postmortem release of PTX3 stored in circulating neutrophils. The morphological changes of neutrophils begin at 6 h following death^19–21^. In this study, the postmortem intervals of the autopsy cases ranged from 5 to 72 h (36.1 ± 15.7 h), which is long enough to cause postmortem changes in neutrophils. There was no relationship between the plasma PTX3 concentration and postmortem intervals, which suggests that the postmortem release of neutrophil-derived PTX3 into the blood occurred simultaneously and immediately after death. Nevertheless, consistent with previous reported clinical data^13–15^, all cases in the coronary thrombi group exhibited a significantly higher plasma PTX3 concentration than the control cases. This finding was observed without being negated by the postmortem release of PTX3 from circulating neutrophils.

In conclusion, the postmortem plasma PTX3 concentration is a useful marker to indicate when death occurred immediately after the onset of fatal ACS. However, the postmortem plasma PTX3 concentration was higher than the clinical reference, which appeared to be caused by a postmortem release from circulating neutrophils. This study first demonstrated that the clinical reference value of plasma PTX3 cannot be applied to postmortem samples. Further postmortem plasma PTX3 data should be collected to aid in the creation of a specific reference value for cadavers.

## Methods

### Autopsy materials

Medico-legal autopsy cases in our department, including ACS, serious infection, asphyxia and immediate death due to fatal injury were investigated. The ACS groups (n = 20 total) were composed of coronary stenosis, coronary thrombi, and myocardial rupture groups. The coronary stenosis group (n = 9) exhibited apparent coronary stenosis, but an absence of coronary thrombi. In the coronary thrombi group (n = 6), thrombi were found in the coronary artery. The myocardial rupture group (n = 5) presented with myocardial rupture following an AMI. No large injuries were evident on these bodies. The serious infection group (n = 10) consisted of one meningitis and two sepsis cases. The control groups (n = 12 total) consisted of six asphyxia cases, including drowning, flail chest, and aspiration and six immediate death cases due to a fatal injury, including cerebral contusion, traumatic myocardial rupture, traumatic aortic rupture, and spinal cord injury. The summary of these groups is presented in Table 1. The cause of death and postmortem interval were determined on the basis of the autopsy findings and the circumstantial evidence in the autopsy documents. A drug screening test was performed on the database with a forensic toxicology library (MassLynx 4.1 TOF Toxicology Database 1, Waters, Milford, MA, USA) using an LC–TOF-MS system (ACQUITY UPLC-Xevo® G2-S QTof, Waters). Plasma samples were prepared from whole heart blood immediately after the autopsy and stored at −20 °C. After routine examinations, the remnants were used for the PTX3 assay. All procedures performed in studies involving human participants were in accordance with the ethical standards of the institutional and/or national research committee and with the 1964 Helsinki declaration and its later amendments or comparable ethical standards. The use of autopsy materials without informed consent was approved by the Ethical Review Board of Kyoto Prefectural University (reference number ERB-C-442).

### Assay

The plasma PTX3 concentration was determined using an ELISA assay kit for human PTX3 (Human Pentraxin3/TSG-14 ELISA System; Perseus Proteomics, Tokyo, Japan)^14^. White blood cell-large cell count (W-LCC) values were determined by an automated hematology analyzer (pocH®-100i, Sysmex, Kobe, Japan) and were considered as the neutrophil counts. The plasma CRP concentration was determined using latex agglutination turbidimetry in LSI Medience Corporation (Tokyo, Japan). Body mass index (BMI) was calculated using the height and weight data measured at autopsy.

### Immunostaining

For PTX3 immunostaining of hemocytes, whole blood from a healthy volunteer or a cadaver was smeared onto a glass slide, dried by cold air for 30 min, and fixed with buffered formalin/acetone (4:6) for 30 s. The blood smear samples were then incubated for 3 h at room temperature with an anti-PTX3 antibody (Perseus Proteomics) diluted at 1:200 and then with biotin-conjugated anti-mouse IgG followed by streptavidin-conjugated alkaline phosphatase each for 30 min (SAB-AP kit, Nichirei Biosciences, Tokyo, Japan). New Fuchsin was used as the chromogenic substrate.

### Statistical analysis

Quantitative data are presented as means ± SD. A Pearson’s product-moment correlation coefficient was used to test the correlation between two variables. All statistical analyses were performed using EZR (ver. 1.11) (Saitama Medical Center, Jichi Medical University)^35^. *P* < 0.05 was considered to be statistically significant.

## Data Availability

All data generated or analysed during this study are included in this published article (and its Supplementary Information files).
